# Group Telegaming Through Immersive Virtual Reality to Improve Mental Health Among Adolescents With Physical Disabilities: Pre- and Posttrial Protocol

**DOI:** 10.2196/42651

**Published:** 2022-10-13

**Authors:** Byron Lai, Drew Davis, Raven Young, Erin Swanson-Kimani, Cynthia Wozow, Kelli Chaviano, James H Rimmer

**Affiliations:** 1 Division of Pediatric Rehabilitation Medicine Department of Pediatrics University of Alabama at Birmingham Birmingham, AL United States; 2 Dean's Office School of Health Professions University of Alabama at Birmingham Birmingham, AL United States

**Keywords:** disability, physical activity, active video gaming, mindfulness

## Abstract

**Background:**

Adolescents with physical disabilities have higher rates of mental health conditions and issues than adolescents without disabilities, and this disparity was exacerbated by the onset of the COVID-19 pandemic. They also have limited access to on-site programs and nearby peers.

**Objective:**

This pilot aims to investigate the potential effects of a low-dose multiplayer virtual reality telegaming program on depression, socialization, and loneliness among a cohort of children with physical disabilities. A secondary aim is to describe feasibility metrics, namely, recruitment and adherence rates and perceived program enjoyment and satisfaction. The tertiary aim is to describe behavioral mechanisms that affect participant adherence and social participation in the classes.

**Methods:**

This study is a single-group pre- and posttest–designed trial. A single cohort of 12 children with physical disabilities will pilot a 1-month program that includes 2 supervised 1-hour sessions per week of group-based exergaming. Participants will complete questionnaires before and after the program. The primary aim measures will include the Children’s Depression Inventory 2 Short Form, a measure of feelings of depression, and the UCLA Loneliness Scale, a measure of both loneliness and social isolation. Secondary aim measures will include three posttest Likert scale questionnaires: perceived program enjoyment, program satisfaction, and satisfaction with multiplayer experiences. At postintervention or dropout, participants will undergo semistructured interviews to identify behavioral mechanisms that underlie participation. Data will be reported descriptively and be supported by *t* tests as appropriate.

**Results:**

Recruitment procedures started in July 2022. All data are expected to be collected by January 2023. Full trial results are expected to be published by March 2023. Secondary analyses of data will be subsequently published.

**Conclusions:**

This trial tests a peer-to-peer virtual reality telegaming program that includes a completely remote enrollment, assessment, and intervention protocol. This program is accessible and short in duration and frequency, allowing it to be integrated into other interventions. Knowledge obtained from this study will inform the development of a larger trial for improving the mental health and well-being of adolescents with physical disabilities.

**Trial Registration:**

ClinicalTrials.gov NCT05259462; https://clinicaltrials.gov/ct2/show/NCT05259462

**International Registered Report Identifier (IRRID):**

PRR1-10.2196/42651

## Introduction

For people with physical disabilities, adolescence is a critical period for school educators and health professionals to provide quality education and health services. In adolescence, people with disabilities build their self-identity and adopt vocational skills and health behaviors that increase the likelihood of living healthy independent lifestyles as they transition into early adulthood [1-3]. Prior to COVID-19, adolescents with disabilities lagged behind in the development of adult life skills [4] and experienced alarmingly higher rates of mental health disorders such as depression and isolation than peers without disabilities [5]. They were also far less likely to engage in social- and health-enhancing physical activities [1,6-8]. Physical activity is a critical behavior for improving gross motor function [9] and health conditions (eg, cardiovascular disease, pain, and fatigue) [10-12], and developing meaningful social relationships with peers in this population.

Disappointingly, reports from the COVID-19 era have found that stress, depression, isolation, and physical activity participation have substantially worsened since the outbreak of COVID-19 among adolescents with disabilities [13-17]. A local report in the southeast United States found that basic needs were met for most of the families [13]. However, 32.7% of adolescents felt down or depressed, 47.5% felt little pleasure in doing things, and 64.4% felt isolated after COVID-19. Moreover, 74.3% reported decreased socialization, and 51.5% reported reduced exercise participation.

In April 2020, one author (BL) and an adapted fitness instructor conducted a home-based community virtual reality program for adolescent members of a fitness facility for people with disabilities. The program included low-cost virtual reality equipment, and people could participate from home during the height of the COVID-19 pandemic amid social distancing mandates, occupancy limits, and facility closures. The program was 1 month in duration and included two 30-minute sessions per week of synchronous group exercise. The program included a total of 10 participants, completed in two intervention waves (n=5 per wave). Since this project was not a research study, the anecdotal resultant themes were *COVID-19–created barriers to physical activity opportunities*; *virtual reality group exercise-filled voids in exercise participation and human connectedness that were created by the COVID-19 pandemic*; and *the virtual reality headset fostered immersion, which increased their motivation to game*. In summary, group virtual reality gaming provided an accessible opportunity for adolescents with disabilities to build new friendships with peers, which enhanced their mental health. There is a need to start confirming these benefits through quantitative investigation, particularly among adolescents living in the Southeast, a geographic region with some of the lowest rates of health care access across the United States (as reported by the United Health Foundation) [18].

This study has 3 purposes. The first purpose is to examine the effects of a home-based virtual reality multiplayer gaming program on depression, socialization, and loneliness among 12 adolescents with physical disabilities (ages 13-19 years). The second purpose is to describe the acceptability of the program as measured by participant adherence, total play time, exercise time, and perceived program satisfaction and enjoyment. The third purpose is to describe behavioral mechanisms that affect participant engagement. We will qualitatively explore participants’ perceptions of barriers and facilitators that affected their adherence and social participation in the classes.

## Methods

### Study Design

This study is a single group pre-to-post design trial that will last 1 year and include 2 waves (n=6 per wave). Participants will be recruited from the medical and billing record databases at the Children’s Hospital, as well as through the collaborative network of the university and an adapted fitness facility for people with disabilities (Lakeshore Foundation, Homewood, Alabama). We plan to recruit a convenience sample of 12 people to satisfy minimum recommendations for a feasibility study that aims to inform sample size considerations for a larger trial [19].

### Ethics Approval

The protocol and informed consent and assent forms were approved by the Institutional Review Board for Human Use of the University (IRB-300008913) on May 23, 2022. Prospective participants and their caregivers will be mailed a consent and assent form, along with the study questionnaires. Participants will be instructed to review the consent and assent forms carefully, sign them, and then complete the study questionnaires. Once completed, they will be instructed to mail the documents back in a return envelope to research staff. Should the participants not want to participate, no action will be needed. Consent and assent documents will be written in English.

### Eligibility Criteria

Eligibility criteria will include self-reported mobility disability (eg, use of a mobility device or presence of a mobility impairment), between the ages of 13-19 years (World Health Organization definition of adolescence and the minimum age of 13 years as recommended by the manufacturer of the virtual reality headset), access to a Wi-Fi internet connection in the home, and a caregiver to support the child if <18 years of age. The exclusion criteria will be as follows: physically active (defined as >150 minutes/week of moderate-to-vigorous intensity exercise), cannot use their arms for exercise or operate the controller buttons using their fingers, and complete blindness or deafness.

### Procedures

Before starting the intervention, participants will be mailed consent/assent forms and baseline surveys. Once the forms are received by research staff, participants will be mailed the intervention equipment. Post intervention (week 5), participants will be mailed another packet of surveys to complete and return to research staff. Participants will then be asked to participate in a one-on-one semistructured interview via phone call or Zoom (Zoom Video Communications, Inc) with the principal investigator, which will be audio recorded and transcribed for qualitative analysis. Participants will receive an electronic US $40 gift card for each packet of surveys returned (total of US $80 for completing the study).

### Home-Based Intervention

The program will last 4 weeks and include two 1-hour sessions per week of supervised peer-to-peer gaming. Further details of the program prescription are shown in Multimedia Appendix 1. Participants will use an Oculus Quest 2 headset to meet peers and 2 coaches (a gaming coach and a mindfulness coach) in an online virtual private party. Each session will include participation in one of several games, including RecRoom (a massive online multiplayer game with countless social and active gaming experiences), VRChat (a game focused on building social relationships), Beat Saber (a rhythmic music-to-movement game), and After the Fall (a cooperative first-person shooter). Each session will include a 5-minute introduction that will include behavior change and mindfulness coaching to promote autonomy, competence, and relatedness through a respectful, cohesive, and positive atmosphere (strategies framed by the self-determination theory [20-22], learned from the mindfulness coaching workshops hosted by the National Center on Health, Physical Activity and Disability). Some of the mindfulness-based strategies will include guided breathing focused exercises, body scanning, meditation, and acceptance of social anxiety and shyness. Game order prescriptions (ie, duration, intensity, time, and type) will be conducted on a *learn-as-you-go* approach based on participants’ preferences.

### Outcomes

Baseline participant characteristics will include age, sex, and ethnicity. Aim 1 measures will include the Children’s Depression Inventory 2 Short Form, a measure of feelings of depression with strong psychometric properties among adolescents with and without disabilities [23,24], and version 3 of the UCLA Loneliness Scale 20 items (UCLA-20) [25-27]. The UCLA-20, version 3, is a measure of both loneliness and social isolation with strong psychometric properties among a variety of age groups and disability groups [27,28]. These measures will be completed at baseline and post intervention (week 5).

Aim 2 will be evaluated through feasibility metrics: recruitment rates, implementation and resource management issues, participant attendance in class (percent of classes attended divided by the total) and outside of class (play time not within the scheduled class sessions), and program satisfaction and enjoyment. Two components of satisfaction will be measured, namely, satisfaction with program delivery and satisfaction with playing with others. Both components will be measured by Likert scale surveys (Multimedia Appendix 2). Survey questions for program delivery will ask participants how satisfied they were with social interactions, online gameplay, and how classes were conducted by the instructors. Survey questions for “playing with others” will ask participants if and how often they played with both peers and people outside of class, and the strength of the friendships or relationships that were created. Overall enjoyment of the program will be measured in a similar manner with a single Likert scale question (Multimedia Appendix 2).

Aim 3 will be measured through one-on-one semistructured interviews of participants’ perceptions of the program. Interviews will be conducted via phone call with the participant and principal investigator (BL). Interviews will last up to 30 minutes and contain 10 general questions, examining barriers and facilitators to adherence, likes and dislikes with the program and equipment, and recommendations to improve the program (Multimedia Appendix 3). General questions will include follow-up questions that probe underlying behavioral mechanisms to participation and perceived benefits to participation. The interviewer (BL) has completed over 400 interviews related to disability and exercise. Interviews will be audio recorded and then transcribed for analysis. The qualitative component will be published in a secondary analysis publication.

### Analyses

The research team’s philosophical assumptions will align with dialectical pluralism [29]. This paradigm posits that the research team should hold separate theoretical perspectives for the quantitative and qualitative methods. The research team will conduct the quantitative component of the study under the conventional positivism perspective, while the qualitative component will be conducted under an interpretivist perspective.

As a preliminary study, the study is not powered for efficacy. Instead, the findings will provide outcome estimates that will inform sample size and design considerations for a larger trial. Descriptive statistics for all quantitative outcomes will include means, SDs, effect sizes, box and whisker plots, and 95% CIs as appropriate. Aim 1 will include *t* tests to compare pre-to-post changes in survey scores. Aim 2 feasibility metrics will be reported in a descriptive manner with no a priori criteria for acceptability.

Aim 3 will be analyzed using a qualitative 6-step process of thematic analysis [30] by two members of the research team. In summary, two analysts will generate codes from segments of the transcribed interviews. The codes will be organized into representative subthemes. The analysts will repeat this process for each transcription. The subthemes will then be merged into higher order themes, which will be reported. The analysts will use a relativist approach of enhancing the quality of the qualitative research findings [31]. First, the research findings will aim to provide a *substantive contribution* [32], that is, the findings will aim to understand how participants interact with the program to inform other investigators who develop similar interventions. Second, *coherence* will be sought by ensuring that qualitative study procedures throughout the project align with the goals of the study [33]. Third, *transparency* will be maintained throughout the study by receiving in-depth feedback from a *critical friend* [34]. The qualitative researchers of the study will scrutinize matters including the theoretical preferences, qualitative procedures, and resultant findings to encourage reflexivity and alternative explanations and interpretations of the data.

## Results

This study was approved by the university institutional review board on May 23, 2022. The study was initiated in February 2022, and the first participant was enrolled in July 2022. Recruitment of the last participant is anticipated in October 2022.

## Discussion

### Anticipated Findings

This study will investigate the preliminary benefits of a home-based mindfulness program that is combined with peer-to-peer virtual reality gaming on mental health among adolescents with disabilities. A program that is beneficial toward depression, anxiety, and isolation, and connects peers remotely at the home using low-cost equipment could greatly benefit this population, which has experienced poorer mental health since the outbreak of the COVID-19 pandemic [5]. The quantitative study component will provide outcomes estimates of the effects of the program on depression, social isolation, and loneliness, and the results will be used to inform sample size considerations for a large clinical trial. The qualitative results will aid in understanding behavioral mechanisms that underlie successful participation, which will help us operationalize implementation procedures in a future trial and explain changes in quantitative outcomes.

In addition to concerns of poor mental health among adolescents with disabilities, health professionals are often concerned with maintaining or improving physical function [35,36] or alleviating and preventing a variety of other health conditions that arise from sedentary behavior (eg, pain, obesity, pressure ulcers, and low bone mineral density) [37]. There is evidence to suggest that physical health concerns can be addressed by combining health-enhancing interventions with the latest immersive virtual reality technology [38,39], but to the best of our knowledge, there is no peer-to-peer group-based intervention for improving mental health. Given the multifaceted nature of health promotion among this population, this study will test a brief low-dose program so that it can be easily integrated within clinical practice and disseminated with other health-enhancing interventions.

### Strengths and Limitations

An innovative component of the study is that it incorporates remote study procedures. Due to difficulties with allocating transportation, a remote study procedure increases accessibility by eliminating the need for on-site visitation. This study has limitations. The sample size is small, and the results will need to be confirmed with larger clinical trials. Additionally, this study requires participants to be able to use the handheld controllers and view the screen of the virtual reality headsets, which may not be appropriate for some people who have functional or visual impairments.

### Conclusions

Should the findings of this study suggest a potential benefit toward some aspects of mental health, the study may discover an innovative and, most importantly, scalable method for addressing mental health among children with physical disabilities.

## Appendix

Multimedia Appendix 1 Intervention plan.

Multimedia Appendix 2 Study survey questions for program satisfaction and enjoyment.

Multimedia Appendix 3 Interview questions.

Multimedia Appendix 4 Peer review report from the Center for Engagement in Disability Health and Rehabilitation Sciences (CEDHARS) Pilot Grant Funding Program: Addressing Health Disparities in Adults or Children with Disabilities - University of Alabama at Birmingham (USA).

## Abbreviations

UCLA-20: UCLA Loneliness Scale 20 items

## Data Availability

The data sets generated or analyzed during this study are available from the corresponding author on reasonable request.
