# Supplementary material for: Group Telegaming Through Immersive Virtual Reality to Improve Mental Health Among Adolescents With Physical Disabilities: Pre- and Posttrial Protocol
Source: JMIR Res Protoc. 2022 Oct 13;11(10):e42651. doi: 10.2196/42651 (PMC9614625; doi:10.2196/42651)
Supplement: Multimedia Appendix 1 [file resprot_v11i10e42651_app1.docx]

| Table 1. Intervention prescription | | | | |
| --- | --- | --- | --- | --- |
|  | Week 1 | Week 2 | Week 3 | Week 4 |
| Frequency (sessions per week) | 2 | 2 | 2 | 2 |
| Intensity (Session 1) | Introductions, set a positive non-judgemental atmosphere, encourage adding friends out of class play | Emphasize cooperation in games, safe communication practices online, and introduce competitive play | Play competitive games, emphasize laughter and provide positive verbal feedback on performance | Play competitive games, emphasize laughter and provide positive verbal feedback on performance |
| Intensity (Session 2) | Encourage comfortable socialization with peers, encourage adding friends and out of class play | Play cooperation and competitive games | Emphasize comfortable socialization with peers, encourage safe socialization with online players | Start with social experiences with peers, discuss the importance of talking with others and making friendships, end with social room with other online players |
| Time | 60 minutes | 60 minutes | 60 minutes | 60 minutes |
| Type | To be determined | To be determined | To be determined | To be determined |
