# Supplementary material for: Group Telegaming Through Immersive Virtual Reality to Improve Mental Health Among Adolescents With Physical Disabilities: Pre- and Posttrial Protocol
Source: JMIR Res Protoc. 2022 Oct 13;11(10):e42651. doi: 10.2196/42651 (PMC9614625; doi:10.2196/42651)
Supplement: Multimedia Appendix 2 [file resprot_v11i10e42651_app2.pdf]

Satisfaction with program delivery

For each question, please circle your best answer choice.

How satisfied were you with the social interaction during virtual reality game play?

1. Very dissatisfied
2. Moderately dissatisfied
3. Neither satisfied nor dissatisfied
4. Moderately satisfied
5. Very satisfied

How satisfied were you with the online group play?

1.Very dissatisfied
2. Moderately dissatisfied
3. Neither satisfied nor dissatisfied
4. Moderately satisfied
5. Very satisfied

How satisfied are you overall with how the classes were conducted by the instructors?

1. Very dissatisfied
2. Moderately dissatisfied
3. Neither satisfied nor dissatisfied
4. Moderately satisfied
5. Very satisfied

Multiplayer feedback survey

1. Did you play with peers outside of class? If yes, circle yes, and how many times per week?

1<br>Yes	2<br>No

If "Yes" above, How many times per week? If "No", skip below to question 2.

1<br>None	2<br>1-2 times per week	3<br>2-3 times per week	4<br>3-4 times per week	5<br>> 4-5 times per week

2. Have you become friends with any of your peers in the group class? If yes, circle "Yes", and describe how strong these friendships are. If "No", skip to question 3.

1<br>Yes	2<br>No

If "Yes" above, describe how strong these friendships are. If "No", skip below to question 3.

1<br>Strong Friendships/ Best Friends	2<br>Friends	3<br>Someone to relate to	4<br>Growing a friendship	5<br>Maybe in the future, but not right now

3. Did you play with other people outside of the class online? If "Yes", circle "Yes", and how many times per week?

1<br>Yes	2<br>No

If “Yes” above, How many times per week? If “No”, skip below to question 4.

1<br>None	2<br>1-2 times per week	3<br>2-3 times per week	4<br>3-4 times per week	5<br>> 4-5 times per week

4. Outside of the group class, did you establish other friendships with other online people?

1<br>Yes	2<br>No

If “Yes” above, describe how strong these friendships are. If “No”, you have completed the multiplayer feedback survey.

1<br>Strong friendships/ Best Friends	2<br>Friends	3<br>Someone to relate to	4<br>Growing a friendship	5<br>Maybe in the future, but not right now

Program Enjoyment Survey

For this question, please circle your best answer choice.

Overall, how enjoyable were the group classes?

1. Very dissatisfied
2. Moderately dissatisfied
3. Neither satisfied nor dissatisfied
4. Moderately satisfied
5. Very satisfied
