# Supplementary material for: Group Telegaming Through Immersive Virtual Reality to Improve Mental Health Among Adolescents With Physical Disabilities: Pre- and Posttrial Protocol
Source: JMIR Res Protoc. 2022 Oct 13;11(10):e42651. doi: 10.2196/42651 (PMC9614625; doi:10.2196/42651)
Supplement: Multimedia Appendix 3 [file resprot_v11i10e42651_app3.pdf]

Interview Questions: (Prompts: “Could you tell me more about that?”, “What caused that issue?”, “Could we possibly help you with that issue, if so, how?”)

1. As an icebreaker, what do you all like to do on your free time?
2. Overall, what were your thoughts about the program?
3. What did you like about the program?
4. What did you dislike about the program (or recommendations to improve it)?
5. From the depression survey, you reported a _________ (increase/decrease/no change) in how much depression you were feeling. Could you tell us about what caused that?
6. From the loneliness scale, you reported a _________ (increase/decrease/no change) in how isolated or lonely you felt. Could you tell us about what caused that?
7. Okay, now I want you to think about the quality of the social interactions you had with other people in the virtual reality program. In the survey, you reported a _____, which means _______. Could you tell us about why you gave that number?
8. Okay, now I want you to think about how satisfied you were about how the program was run by the coaches. In the survey, you reported a _____, which means ______. Could you tell us more about that?
9. Alright, last question. You attended ______ (some [<25%]/many [26-74%]/most [74%-99%]/all) of the group classes.
    a. What were some reasons you could not make it to class (if applicable)?
    b. What motivated you to attend the classes?
10. Do you have any recommendations for improving the group program?
