# Supplementary material for: Group Telegaming Through Immersive Virtual Reality to Improve Mental Health Among Adolescents With Physical Disabilities: Pre- and Posttrial Protocol
Source: JMIR Res Protoc. 2022 Oct 13;11(10):e42651. doi: 10.2196/42651 (PMC9614625; doi:10.2196/42651)
Supplement: Multimedia Appendix 4 [file resprot_v11i10e42651_app4.pdf]

Application Review Form

Thank you in advance for helping to review applications received for the CEDHARS Pilot: Addressing Health Disparities in Adults or Children with Disabilities. To access the proposal, click on the "Download Proposal" icon below. Please read, assess and score the application based on the parameters listed below.

Notes about this form: Chrome is the preferred browser. If you would like to begin filling out this review and return to it later, please scroll to the bottom of this form and click 'Save and Return Later'. Then, please follow the instructions provided in the pop-up windows. Be sure to save your Return Code and send yourself an email containing the link to this partially-completed review form. You will need both the link and the Return Code to re-access this partially completed review form. If the email does not appear in your Inbox, please check your Junk folder.

Response was added on 01/15/2022 10:29am.

Reviewer: 1

Statement of Confidentiality and Intellectual Property Protection

By agreeing to provide this review, you agree not to disclose or discuss the application or proposal, or associated materials made available to you with anyone other than the review administrator. You further agree not to use information contained in an application or proposal for your personal benefit or to make such information available for the personal benefit of any other individual or organization.
I agree to uphold confidentiality regarding this proposal:

⊗ Yes
◯ No

Proposal Details

Proposal Title:

Improving Social Isolation and Loneliness Among Children with Physical Disabilities Through Group-Based Online Virtual Reality Gaming

---

Principal Investigator

Name: Byron Lai
Degrees: MS, PhD
Institution: University of Alabama at Birmingham
Department: Pediatrics
Rank: Assistant Professor

You can download the proposal assigned to this review by clicking on the link below.

Download Proposal

Review

- NIH Scoring Guide -

A score of 5 is a good, medium-impact application.

The entire scale (1-9) should always be considered.

An application does not need to be strong in all categories to be judged likely to have major impact.

NIH Scoring Guide

Score	Impact	Descriptor	Additional Guidance on Strength/Weakness
1.0	High	Exceptional	Exceptionally strong with essentially no weaknesses
2.0		Outstanding	Extremely strong with negligible weaknesses
3.0		Excellent	Very strong with only some minor weaknesses
4.0	Medium	Very Good	Strong but with numerous minor weaknesses
5.0		Good	Strong but with at least one moderate weakness
6.0		Satisfactory	Some strengths but also some moderate weaknesses
7.0	Low	Fair	Some strengths but with at least one major weakness
8.0		Marginal	A few strengths and a few major weaknesses
9.0		Poor	Very few strengths and numerous major weaknesses

Does the proposal describe a project that aligns with the mission of the funding opportunity?

⊗ Yes
◯ No
◯ Somewhat

Please explain your answer with comments that are helpful to the applicant:

Yes - focus on mental health of adolescents with disabilities. PI is junior faculty member.

Significance and Relevance

Does the project address an important problem or critical barrier to progress in the field? Is the prior research that serves as the key support for the proposed project rigorous? If the aims are achieved, will scientific knowledge, technical capability, or clinical practice be improved?
Please provide a score based on the NIH's Scoring System:

◯ 1- Exceptional
◯ 2- Outstanding
⊗ 3- Excellent
◯ 4- Very Good
◯ 5- Good
◯ 6- Satisfactory
◯ 7- Fair
◯ 8- Marginal
◯ 9- Poor

Please explain your score with comments on strengths and weaknesses that are helpful to the applicant.

Yes, mental health and mood of adolescents with disabilities is significant public health problem. Efforts to reduce negative health outcomes are quite worthy of study, and this study uses readily-available technology to evaluate a prevention strategy. Prior research is compelling. Biggest concern here is whether the proposed research offers any extension of existing and ongoing research. It seems to replicate what is already known and the additional contribution of this study is unclear.

Investigator(s)

Are the PI and other investigator(s) well suited to the project? If Early Stage Investigators or New Investigators, do they have appropriate experience and training? If established, have they demonstrated an ongoing record of accomplishments that have advanced their field(s)? If mentorship is proposed, does the mentorship plan ensure accomplishment of the project aims?
Please provide a score based on the NIH's Scoring System:

⊗ 1- Exceptional
◯ 2- Outstanding
◯ 3- Excellent
◯ 4- Very Good
◯ 5- Good
◯ 6- Satisfactory
◯ 7- Fair
◯ 8- Marginal
◯ 9- Poor

Please explain your score with comments on strengths and weaknesses that are helpful to the applicant.

PI Dr. Lai is very well suited to the project. Dr. Lai's previous research leads directly into this proposal and his research trajectory is very impressive for someone in their first year as a UAB faculty member. Dr. Lai is well prepared to start writing R21s, R01s, and similar federal grants in the next year or two, building from pilot funding like this opportunity. No mentorship is explicitly mentioned, but it seems likely that it would occur.

Innovation

Does the proposed work seek to shift current research or clinical practice paradigms by utilizing (refining, improving or newly applying) novel theoretical concepts, approaches or methodologies, instrumentation, or interventions? Are they novel to one field of research or novel in a broad sense? Please provide a score based on the NIH's Scoring System:

◯ 1- Exceptional
◯ 2- Outstanding
◯ 3- Excellent
◯ 4- Very Good
⊗ 5- Good
◯ 6- Satisfactory
◯ 7- Fair
◯ 8- Marginal
◯ 9- Poor

Please explain your score with comments on strengths and weaknesses that are helpful to the applicant.

Innovation is mixed. The use of VR technology is impressive, but not highly unusual anymore. The use of telehealth type strategies also is impressive but not highly unusual. The innovation of this research seems modest at best. It seems to grow directly from previous research by the PI and other investigators nationwide, and it is unclear whether an additional pilot study is required. It feels like a large clinical trial would be the logical next step. Data should be available to estimate sample size for such a trial, and it is unclear to me whether this particular pilot study is actually needed.

Approach

Are the overall strategy, methodology, and analyses well-reasoned and appropriate to accomplish the specific aims of the project? Are potential problems, alternative strategies, and benchmarks for success presented? If the project is in the early stages of development, will the strategy establish feasibility and will particularly risky aspects be managed? Given the experience of the research team, is this feasible for the proposed time frame? If the project involves human subjects and/or clinical research, are the plans to address the protection of human subjects from research risks, and inclusion (or exclusion) of individuals on the basis of sex/gender, race and ethnicity, and age justified in terms of the scientific goals and research strategy proposed?
Please provide a score based on the NIH's Scoring System:

◯ 1- Exceptional
◯ 2- Outstanding
◯ 3- Excellent
⊗ 4- Very Good
◯ 5- Good
◯ 6- Satisfactory
◯ 7- Fair
◯ 8- Marginal
◯ 9- Poor

Please explain your score with comments on strengths and weaknesses that are helpful to the applicant.

Approach is reasonable; a straightforward pre-post research design with both quantitative and qualitative components given the small sample size of 12. It would establish feasibility for larger studies, although I question whether previous and ongoing work by the PI accomplishes the same goal. Human subjects issues are not directly addressed but do not seem problematic. Inclusion of both boys and girls, and people of diverse racial/ethnic backgrounds is also not addressed but likely to be appropriate. Focus on adolescents is appropriate and logical. I have a few thoughts about the approach that the PI might consider if the study is funded.

- Opening paragraph of specific aims focuses on children but this proposal is about adolescents. Strange inconsistency
- Details of how the sessions will be supervised/guided are a bit fuzzy to me. Why are two coaches required (extra manpower and cost for long-term implementation)?
- Age range of 13-19 is very wide. Raises concerns about developmental issues (e.g., cognition) as well as social ones (e.g., will an 18- or 19-year-old feel socially connected to a 13- or 14-year-old? And vice versa. I am doubtful; would recommend careful attention to developmental issues and the age range used, especially in larger NIH-funded trial)
- End of specific aims calls this a brief, low-dose program. How so? Strikes me as fairly long and fairly high dose. It also talks about incorporated this into an exercise trial, which confuses me. Isn't this an exercise trial itself? And why incorporate this into something larger, introducing potential bias? Both issues should be explained/addressed.
- Not sure focus on adolescents living in the Southeast is something to advertise. Generalizability across the country would be nice.
- BIG concern: no control group. The design lends itself perfectly to a control group, in that Wave 2 could be assessed during Wave 1 to provide some control measures without even adding additional participants. To be compelling pilot research for an R01, I think a control group would be valuable and recommend trying to incorporate it into this design if possible.
- Why have questionnaires completed by mail? That introduces concerns that parents might see (and bias) answers, especially for the younger participants. It also feels quite antiquated in today's world. Look into using Qualtrics or Redcap to administer the questionnaires securely and remotely over the internet.
- Very little details are provided about the thematic analysis of qualitative data. Does the research team have appropriate expertise to do this correctly?

One last point: it is unclear to me what ongoing research is happening that the PI is leading, and whether this proposal overlaps (or does not overlap) with those projects.

Human Subject or Animal Protections

Are there any potential human subjects and/or animal protection concerns?

◯ Yes
⊗ No

Budget & Timeline

Are there any concerns with the budget or timeline?

⊗ Yes
◯ No

Please explain:

Timeline could be altered somewhat. Preparation and IRB approval could happen within 3-4 months, not 5. Manuscript preparation could happen within 2 months, not 4. Recruitment and assessment might take longer than proposed. But the full project should be feasible to accomplish in a year.

Extramural Competitiveness:

Is it likely that successful completion of this project will provide preliminary data that will lead to a subsequent NIH (or equivalent) grant?

⊗ Yes
◯ No

Overall Impact Score

Assess the likelihood the project may progress to a sustained, powerful influence on the research field(s) involved. Your score should represent a global view; an application does not need to be strong in all categories to be judged likely to have major scientific impact, and the score does not need to be a mathematical reflection of the sections above.

◯ 1- Exceptional
◯ 2- Outstanding
⊗ 3- Excellent
◯ 4- Very Good
◯ 5- Good
◯ 6- Satisfactory
◯ 7- Fair
◯ 8- Marginal
◯ 9- Poor

Please explain your score with comments on strengths and weaknesses that are helpful to the applicant.

Strong study by strong investigator. Major concern is whether this is replicative of previous and ongoing research by the PI and others. Is this sort of pilot really needed, or could the field move onto larger clinical trials? Other concerns are lack of control group, need for 2 coaches (a problem for implementation), and lack of details on training program and data analysis (including qualitative analysis).

Application Review Form

Thank you in advance for helping to review applications received for the CEDHARS Pilot: Addressing Health Disparities in Adults or Children with Disabilities. To access the proposal, click on the "Download Proposal" icon below. Please read, assess and score the application based on the parameters listed below.

Notes about this form: Chrome is the preferred browser. If you would like to begin filling out this review and return to it later, please scroll to the bottom of this form and click 'Save and Return Later'. Then, please follow the instructions provided in the pop-up windows. Be sure to save your Return Code and send yourself an email containing the link to this partially-completed review form. You will need both the link and the Return Code to re-access this partially completed review form. If the email does not appear in your Inbox, please check your Junk folder.

Response was added on 01/17/2022 11:46am.

Reviewer: 2

Statement of Confidentiality and Intellectual Property Protection

By agreeing to provide this review, you agree not to disclose or discuss the application or proposal, or associated materials made available to you with anyone other than the review administrator. You further agree not to use information contained in an application or proposal for your personal benefit or to make such information available for the personal benefit of any other individual or organization.
I agree to uphold confidentiality regarding this proposal:

⊗ Yes
◯ No

Proposal Details

Proposal Title:

Improving Social Isolation and Loneliness Among Children with Physical Disabilities Through Group-Based Online Virtual Reality Gaming

---

Principal Investigator

Name: Byron Lai
Degrees: MS, PhD
Institution: University of Alabama at Birmingham
Department: Pediatrics
Rank: Assistant Professor

You can download the proposal assigned to this review by clicking on the link below.

Download Proposal

Review

- NIH Scoring Guide -

A score of 5 is a good, medium-impact application.

The entire scale (1-9) should always be considered.

An application does not need to be strong in all categories to be judged likely to have major impact.

NIH Scoring Guide

Score	Impact	Descriptor	Additional Guidance on Strength/Weakness
1.0	High	Exceptional	Exceptionally strong with essentially no weaknesses
2.0		Outstanding	Extremely strong with negligible weaknesses
3.0		Excellent	Very strong with only some minor weaknesses
4.0	Medium	Very Good	Strong but with numerous minor weaknesses
5.0		Good	Strong but with at least one moderate weakness
6.0		Satisfactory	Some strengths but also some moderate weaknesses
7.0	Low	Fair	Some strengths but with at least one major weakness
8.0		Marginal	A few strengths and a few major weaknesses
9.0		Poor	Very few strengths and numerous major weaknesses

Does the proposal describe a project that aligns with the mission of the funding opportunity?

⊗ Yes
◯ No
◯ Somewhat

Please explain your answer with comments that are helpful to the applicant:

The proposal clearly meets many of the state priority areas for the CEDHARS P&F program, specifically "Interventions aimed at reducing social isolation/loneliness", but is relevant to other priority areas as well.

Significance and Relevance

Does the project address an important problem or critical barrier to progress in the field? Is the prior research that serves as the key support for the proposed project rigorous? If the aims are achieved, will scientific knowledge, technical capability, or clinical practice be improved?
Please provide a score based on the NIH's Scoring System:

◯ 1- Exceptional
⊗ 2- Outstanding
◯ 3- Excellent
◯ 4- Very Good
◯ 5- Good
◯ 6- Satisfactory
◯ 7- Fair
◯ 8- Marginal
◯ 9- Poor

Please explain your score with comments on strengths and weaknesses that are helpful to the applicant.

The proposed project has the potential to address two key problems for adolescents with PD; loneliness/social isolation and reducing sedentary time/increasing physical activity.

The use of the (relatively inexpensive) Oculus Quest device to achieve these goals is significant (and innovative).

Investigator(s)

Are the PI and other investigator(s) well suited to the project? If Early Stage Investigators or New Investigators, do they have appropriate experience and training? If established, have they demonstrated an ongoing record of accomplishments that have advanced their field(s)? If mentorship is proposed, does the mentorship plan ensure accomplishment of the project aims?
Please provide a score based on the NIH's Scoring System:

⊗ 1- Exceptional
◯ 2- Outstanding
◯ 3- Excellent
◯ 4- Very Good
◯ 5- Good
◯ 6- Satisfactory
◯ 7- Fair
◯ 8- Marginal
◯ 9- Poor

Please explain your score with comments on strengths and weaknesses that are helpful to the applicant.

Dr. Lai is well-suited to conduct the proposed research.
This a nice extension of his previous CEDHARS pilot project and feasibility work using VR in youth with Spina Bifida.

Innovation

Does the proposed work seek to shift current research or clinical practice paradigms by utilizing (refining, improving or newly applying) novel theoretical concepts, approaches or methodologies, instrumentation, or interventions? Are they novel to one field of research or novel in a broad sense? Please provide a score based on the NIH's Scoring System:

⊗ 1- Exceptional
◯ 2- Outstanding
◯ 3- Excellent
◯ 4- Very Good
◯ 5- Good
◯ 6- Satisfactory
◯ 7- Fair
◯ 8- Marginal
◯ 9- Poor

Please explain your score with comments on strengths and weaknesses that are helpful to the applicant.

Use of the Oculus Quest device is highly innovative. The immersive nature of the VR increases the likelihood of both improving issues related loneliness/social isolation and increasing engagement in health-promoting exercise.

Approach

Are the overall strategy, methodology, and analyses well-reasoned and appropriate to accomplish the specific aims of the project? Are potential problems, alternative strategies, and benchmarks for success presented? If the project is in the early stages of development, will the strategy establish feasibility and will particularly risky aspects be managed? Given the experience of the research team, is this feasible for the proposed time frame? If the project involves human subjects and/or clinical research, are the plans to address the protection of human subjects from research risks, and inclusion (or exclusion) of individuals on the basis of sex/gender, race and ethnicity, and age justified in terms of the scientific goals and research strategy proposed?
Please provide a score based on the NIH's Scoring System:

◯ 1- Exceptional
◯ 2- Outstanding
⊗ 3- Excellent
◯ 4- Very Good
◯ 5- Good
◯ 6- Satisfactory
◯ 7- Fair
◯ 8- Marginal
◯ 9- Poor

Please explain your score with comments on strengths and weaknesses that are helpful to the applicant.

Overall, the approach is strong and feasible within the given time frame.

Minor weaknesses include:
* Lack of control or usual care condition, but understandable given the goals and scope of the proposal
* Not clear how Aim 2 measures related to exercise time and total play time (in and outside of classes) are measured.

Human Subject or Animal Protections

Are there any potential human subjects and/or animal protection concerns?

◯ Yes
⊗ No

Budget & Timeline

Are there any concerns with the budget or timeline?

⊗ Yes
◯ No

Please explain:

Minor: Text states $60 stipend for each returned packet (up to $120 total per participant), but budget only includes enough funds for $40 ($80 total).

Extramural Competitiveness:

Is it likely that successful completion of this project will provide preliminary data that will lead to a subsequent NIH (or equivalent) grant?

⊗ Yes
◯ No

Overall Impact Score

Assess the likelihood the project may progress to a sustained, powerful influence on the research field(s) involved. Your score should represent a global view; an application does not need to be strong in all categories to be judged likely to have major scientific impact, and the score does not need to be a mathematical reflection of the sections above.

◯ 1- Exceptional
⊗ 2- Outstanding
◯ 3- Excellent
◯ 4- Very Good
◯ 5- Good
◯ 6- Satisfactory
◯ 7- Fair
◯ 8- Marginal
◯ 9- Poor

Please explain your score with comments on strengths and weaknesses that are helpful to the applicant.

Through the completion of this proposal and with existing data from his previous CEDHARS-supported work, Dr. Lai should be well-positioned for extramural federal research support.

Application Review Form

Thank you in advance for helping to review applications received for the CEDHARS Pilot: Addressing Health Disparities in Adults or Children with Disabilities. To access the proposal, click on the "Download Proposal" icon below. Please read, assess and score the application based on the parameters listed below.

Notes about this form: Chrome is the preferred browser. If you would like to begin filling out this review and return to it later, please scroll to the bottom of this form and click 'Save and Return Later'. Then, please follow the instructions provided in the pop-up windows. Be sure to save your Return Code and send yourself an email containing the link to this partially-completed review form. You will need both the link and the Return Code to re-access this partially completed review form. If the email does not appear in your Inbox, please check your Junk folder.

Response was added on 01/31/2022 10:35am.

Reviewer: 3

Statement of Confidentiality and Intellectual Property Protection

By agreeing to provide this review, you agree not to disclose or discuss the application or proposal, or associated materials made available to you with anyone other than the review administrator. You further agree not to use information contained in an application or proposal for your personal benefit or to make such information available for the personal benefit of any other individual or organization.
I agree to uphold confidentiality regarding this proposal:

⊗ Yes
◯ No

Proposal Details

Proposal Title:

Improving Social Isolation and Loneliness Among Children with Physical Disabilities Through Group-Based Online Virtual Reality Gaming

Principal Investigator

Name: Byron Lai
Degrees: MS, PhD
Institution: University of Alabama at Birmingham
Department: Pediatrics
Rank: Assistant Professor

You can download the proposal assigned to this review by clicking on the link below.

Download Proposal

Review

- NIH Scoring Guide -

A score of 5 is a good, medium-impact application.

The entire scale (1-9) should always be considered.

An application does not need to be strong in all categories to be judged likely to have major impact.

NIH Scoring Guide			
Score	Impact	Descriptor	Additional Guidance on Strength/Weakness
1.0	High	Exceptional	Exceptionally strong with essentially no weaknesses
2.0		Outstanding	Extremely strong with negligible weaknesses
3.0		Excellent	Very strong with only some minor weaknesses
4.0	Medium	Very Good	Strong but with numerous minor weaknesses
5.0		Good	Strong but with at least one moderate weakness
6.0		Satisfactory	Some strengths but also some moderate weaknesses
7.0	Low	Fair	Some strengths but with at least one major weakness
8.0		Marginal	A few strengths and a few major weaknesses
9.0		Poor	Very few strengths and numerous major weaknesses

Does the proposal describe a project that aligns with the mission of the funding opportunity?

⊗ Yes
◯ No
◯ Somewhat

Please explain your answer with comments that are helpful to the applicant:

The proposal aligns well with the CEDHARS RFP as it aims to improve depression and socialization among children with movement disabilities by delivering a novel VR-based, group exercise-gaming intervention.

Significance and Relevance

Does the project address an important problem or critical barrier to progress in the field? Is the prior research that serves as the key support for the proposed project rigorous? If the aims are achieved, will scientific knowledge, technical capability, or clinical practice be improved?
Please provide a score based on the NIH's Scoring System:

⊗ 1- Exceptional
◯ 2- Outstanding
◯ 3- Excellent
◯ 4- Very Good
◯ 5- Good
◯ 6- Satisfactory
◯ 7- Fair
◯ 8- Marginal
◯ 9- Poor

Please explain your score with comments on strengths and weaknesses that are helpful to the applicant.

The project proposal is highly significant to a current unmet need. The project seeks to overcome barriers and limitations on social interactions and exercise for children with movement disabilities. This problem has been exacerbated over the past year or two due to increased isolation from pandemic reactions. The aims will serve to begin validating the use of an emerging, low-cost VR system for group ever-gaming to improve social isolation and depression in children with movement disabilities. If the aims are achieved then the findings can be used for larger clinical trials, to support grant applications, and to prescribe electronic social-exer-gaming interventions in children with movement disabilities.

Investigator(s)

Are the PI and other investigator(s) well suited to the project? If Early Stage Investigators or New Investigators, do they have appropriate experience and training? If established, have they demonstrated an ongoing record of accomplishments that have advanced their field(s)? If mentorship is proposed, does the mentorship plan ensure accomplishment of the project aims?
Please provide a score based on the NIH's Scoring System:

⊗ 1- Exceptional
◯ 2- Outstanding
◯ 3- Excellent
◯ 4- Very Good
◯ 5- Good
◯ 6- Satisfactory
◯ 7- Fair
◯ 8- Marginal
◯ 9- Poor

Please explain your score with comments on strengths and weaknesses that are helpful to the applicant.

The early stage PI has excellent training and the appropriate background to conduct the studies. The PI has in fact piloted many facets of the work while working between Lakeshore and Children's Hospital. The early data and experimental interventions explored thus far by the PI instills confidence that the tasks laid out by the proposal will be accomplished.

Innovation

Does the proposed work seek to shift current research or clinical practice paradigms by utilizing (refining, improving or newly applying) novel theoretical concepts, approaches or methodologies, instrumentation, or interventions? Are they novel to one field of research or novel in a broad sense? Please provide a score based on the NIH's Scoring System:

⊗ 1- Exceptional
◯ 2- Outstanding
◯ 3- Excellent
◯ 4- Very Good
◯ 5- Good
◯ 6- Satisfactory
◯ 7- Fair
◯ 8- Marginal
◯ 9- Poor

Please explain your score with comments on strengths and weaknesses that are helpful to the applicant.

While VR-based therapies have been implemented across a variety of medical/clinical interventions, there is little know with respect to how to implement social-exer-gaming applications in children with movement disabilities. In addition, the ability to use low-cost VR systems makes the impact of the findings potentially scalable. There are several novel aspects of the experimental intervention planned by the project.

Approach

Are the overall strategy, methodology, and analyses well-reasoned and appropriate to accomplish the specific aims of the project? Are potential problems, alternative strategies, and benchmarks for success presented? If the project is in the early stages of development, will the strategy establish feasibility and will particularly risky aspects be managed? Given the experience of the research team, is this feasible for the proposed time frame? If the project involves human subjects and/or clinical research, are the plans to address the protection of human subjects from research risks, and inclusion (or exclusion) of individuals on the basis of sex/gender, race and ethnicity, and age justified in terms of the scientific goals and research strategy proposed?
Please provide a score based on the NIH's Scoring System:

◯ 1- Exceptional
⊗ 2- Outstanding
◯ 3- Excellent
◯ 4- Very Good
◯ 5- Good
◯ 6- Satisfactory
◯ 7- Fair
◯ 8- Marginal
◯ 9- Poor

Please explain your score with comments on strengths and weaknesses that are helpful to the applicant.

The overall approach of the project is outstanding. The timeline is appropriate. The experimental interventions are low-risk and there is no concern from a human subjects research perspective. The major suggestion offered to the PI for improving the study, would be to use other instruments in addition to the depression scale to quantify outcomes. Since improving social functioning and satisfaction are desired outcomes of the intervention, the PI should include scales for assessing the quality of social interactions and general life satisfaction in children. For example, one may wish to use a modified Quality of Life survey similar to the SF-36 adjusted for children to assess multiple areas of impact beyond depression. Even if one were to use simple Likert scales for assessing the quality of social interactions rated by children and/or parents/gaurdian observers this would be a increased value proposition for the study. It would allow the PI to take a more quantifiable look at the data to subsequently support or optimize the interventions.

Human Subject or Animal Protections

Are there any potential human subjects and/or animal protection concerns?

◯ Yes
⊗ No

Budget & Timeline

Are there any concerns with the budget or timeline?

◯ Yes
⊗ No

Extramural Competitiveness:

Is it likely that successful completion of this project will provide preliminary data that will lead to a subsequent NIH (or equivalent) grant?

⊗ Yes
◯ No

Overall Impact Score

Assess the likelihood the project may progress to a sustained, powerful influence on the research field(s) involved. Your score should represent a global view; an application does not need to be strong in all categories to be judged likely to have major scientific impact, and the score does not need to be a mathematical reflection of the sections above.

⊗ 1- Exceptional
◯ 2- Outstanding
◯ 3- Excellent
◯ 4- Very Good
◯ 5- Good
◯ 6- Satisfactory
◯ 7- Fair
◯ 8- Marginal
◯ 9- Poor

Please explain your score with comments on strengths and weaknesses that are helpful to the applicant.
